# Phenolic Extracts from Extra Virgin Olive Oils Inhibit Dipeptidyl Peptidase IV Activity: In Vitro, Cellular, and In Silico Molecular Modeling Investigations

**DOI:** 10.3390/antiox10071133

**Published:** 2021-07-16

**Authors:** Carmen Lammi, Martina Bartolomei, Carlotta Bollati, Lorenzo Cecchi, Maria Bellumori, Emanuela Sabato, Vistoli Giulio, Nadia Mulinacci, Anna Arnoldi

**Affiliations:** 1 Department of Pharmaceutical Sciences, University of Milan, 20133 Milan, Italy; martina.bartolomei@unimi.it (M.B.); carlotta.bollati@unimi.it (C.B.); emanuela.sabato@studenti.unimi.it (E.S.); giulio.vistoli@unimi.it (V.G.); anna.arnoldi@unimi.it (A.A.); 2 Department of Neuroscience, Psychology, Drug and Child Health, Pharmaceutical and Nutraceutical Section, University of Florence, 50019 Florence, Italy; lo.cecchi@unifi.it (L.C.); maria.bellumori@unifi.it (M.B.); nadia.mulinacci@unifi.it (N.M.)

**Keywords:** Caco-2 cells, DPP-IV, EVOO, phenolic extracts, secoiridoids

## Abstract

Two extra virgin olive oil (EVOO) phenolic extracts (BUO and OMN) modulate DPP-IV activity. The in vitro DPP-IV activity assay was performed at the concentrations of 1, 10, 100, 500, and 1000 μg/mL, showing a dose-dependent inhibition by 6.8 ± 1.9, 17.4 ± 6.1, 37.9 ± 2.4, 57.8 ± 2.9, and 81 ± 1.4% for BUO and by 5.4 ± 1.7, 8.9 ± 0.4, 28.4 ± 7.2, 52 ± 1.3, and 77.5 ± 3.5% for OMN. Moreover, both BUO and OMN reduced the DPP-IV activity expressed by Caco-2 cells by 2.9 ± 0.7, 44.4 ± 0.7, 61.2 ± 1.8, and 85 ± 4.2% and by 3 ± 1.9, 35 ± 9.4, 60 ± 7.2, and 82 ± 2.8%, respectively, at the same doses. The concentration of the most abundant and representative secoiridoids within both extracts was analyzed by nuclear magnetic resonance (^1^H-NMR). Oleuropein, oleacein, oleocanthal, hydroxytyrosol, and tyrosol, tested alone, reduced the DPP-IV activity, with IC_50_ of 472.3 ± 21.7, 187 ± 11.4, 354.5 ± 12.7, 741.6 ± 35.7, and 1112 ± 55.6 µM, respectively. Finally, in silico molecular docking simulations permitted the study of the binding mode of these compounds.

## 1. Introduction

Dipeptidyl-peptidase-IV (DPP-IV), a serine exopeptidase expressed on the surface of most cell types, is able to cleave Xaa-proline or Xaa-alanine dipeptides from the *N*-terminus of polypeptides. Among all DPP-IV substrates, the most widely investigated are glucagon-like peptide 1 (GLP-1) and glucose-dependent insulinotropic polypeptide (GIP), two incretins playing essential roles in controlling post-prandial glycemia [1,2]. In more detail, incretins such as GLP-1 and GIP are secreted by the distal small intestine in response to its stimulation and bind receptors in the endocrine pancreas, thus eliciting insulin secretion and lowering post-prandial blood glucose [3,4,5]. Notably, both hormones are rapidly inactivated by DPP-IV, whose activation is increased by oxidative stress [6]. Hence, its inhibition is an established glucose-lowering therapy in type 2 diabetes [7,8]. Post-prandial glucose has been associated with a higher incidence of cardiovascular events in patients with [9] and without [10] diabetes. Indeed, reducing post-prandial glycemia and the lipid profile could have a positive impact on the progression of atherosclerosis [11].

Observational and interventional studies consistently demonstrated a potentially beneficial effect of EVOO consumption on the atherosclerotic process and diabetes [12,13,14,15]. However, the underlying mechanism is still unknown, and a substantial gap of information exists regarding the mechanism of action through which EVOO modulates glycemia. Recently, it was demonstrated that the supplementation of a meal with 10 g EVOO had positive effects on postprandial glycemic profile, due to an increase in incretins concomitantly with a decrease in DPP-IV protein levels and therefore in activity in a group of healthy subjects that consumed a meal supplemented with 10 g of corn oil [12]. Other evidence indicates that oleuropein has similar effects on incretins (GLP-1 and GIP) and the glycemic profile of healthy subjects, suggesting that oleuropein may be the major phenolic component within EVOO responsible for incretin upregulation through the control of DPP-IV protein-level modulation [16].

Based on these considerations, the assessment of the mechanism of action through which EVOO extracts may directly modulate the DPP-IV activity was carried out using in vitro and cellular fluorescent assays. The present investigation was conducted on two phenolic extracts previously obtained from two different cultivars [17]: one from the EVOO of the cultivar Frantoio cultivated in Tuscany (Italy), named BUO, and the other from the cultivar Coratina cultivated in Apulia (Italy), named OMN. Both extracts result in cholesterol-lowering activity [17] in human hepatic HepG2 cells and antioxidant activity [18] in intestinal Caco-2 and HepG2 cells. Phenol characterization, as reported in a preceding paper, was performed by applying the official method (International Olive Council [19]) for quantifying the total phenol content, and a validated hydrolytic method was used to evaluate the total content of hydroxytyrosol and tyrosol as the sum of free and bound forms [20]. The analysis of BUO and OMN extracts highlighted that the total phenolic contents of the two extracts are significantly different [17]. Hence, in order to foster and better highlight the advantageous of EVOO phytocomplex against diabetes, the health-promoting effects of the BUO and OMN extracts were investigated by evaluating their potential hypoglycemic effect through the modulation of DPP-IV activities. In more detail, the first objective of the study was the measurement of the concentration of the secoiridoids oleuropein, oleacein, oleocanthal, and ligstroside aglycone in the two EVOO extracts BUO and OMN by proton nuclear magnetic resonance (^1^H-NMR) analysis. The second objective was the assessment of the DPP-IV inhibitory activity of the two extracts and of the pure standards of oleuropein, oleacein, oleocanthal, hydroxytyrosol, and tyrosol, which are the most abundant and representative compounds within the extracts, by a biochemical assay. Finally, for a further reinforcement of the study, the third objective was an investigation of the possible binding of these compounds to DPIV by in silico molecular docking simulations.

## 2. Materials and Methods

### 2.1. Chemicals

All reagents are commercially available. More details are reported in the Supplementary Material.

### 2.2. Preparation of the BUO and OMN Samples and Quantification of the Main Secoiridoids

The EVOO extracts were prepared as previously reported [17]: the extracts were prepared starting from 5 mL of oil, obtaining a dry weight of 10 mg and 5.7 mg for BUO and OMN, respectively. Their total phenolic content and total hydroxytyrosol and tyrosol were determined applying to validated methods [17].

To evaluate the concentration of oleacein, oleuropein, oleocanthal, and ligstroside aglycone, the ^1^H NMR spectra of BUO and OMN samples were registered by a 400 MHz instrument Advance 400 (Bruker, Bremen, Germany). The extracts were dissolved in 1 mL of CDCl_3_, and 1 µL of the solution of the internal standard (maleic acid 30 mg/mL in CH_3_CN) was added. According to our previous studies [21], to reference guidelines for quantitation by NMR [22], and to Karkoula et al. [23], the evaluation of oleacein, oleocanthal, oleuropein aglycone, and ligstroside aglycone in their monoaldehyde forms was done applying the following formula:

*C(%) = I_CHO_/I_mal_ × N_mal_/N_CHO_ × MW_X_/MW_mal_ × W_mal_/W_sample_ × P_mal_* (1)

*C(%)*, concentration of each of the four secoiridoid aldehydes.
*I_mal_*, integral of 2 protons of the ISTD, maleic acid.
*I_CHO_*, integral area of the proton signal of the aldehyde of each secoiridoid.
*N_CHO_*, the number of the aldehyde proton of each secoiridoid.
*N_mal_*, the number of the protons of maleic acid.
*MW_X_*, the molecular weight of each secoiridoid (oleacein 320 g/moL, oleuropein aglycone 378 g/moL, oleocanthal 304 g/moL, and ligstroside aglycone 362 g/moL).
*MW_mal_*, the mw of ISTD maleic acid, 116.1 g/moL.
*W_mal_*, weight in mg of maleic acid.
*W_sample_*, dry weight in mg of BUO or OMN.
*P_mal_*, purity grade of maleic acid.

### 2.3. In Vitro DPP-IV Activity Assay

The DPP-IV enzyme was provided by Cayman Chemicals (Ann Arbor, MI, USA), while the DPP-IV substrate (Gly-Pro-amido-4-methylcoumarin hydrobromide (Gly-Pro-AMC)) was from AnaSpec Inc. (Freemont, CA, USA). The experiments were carried out in triplicate in a half-volume 96-well solid plate (white) following conditions previously optimized [24]. More details are available in the Supplementary Material.

### 2.4. Cell Culture

Caco-2 cells, obtained from INSERM (Paris, France), were routinely sub-cultured following a previously optimized protocol [25]. More details are available in the Supplementary Material.

### 2.5. In Situ DPP-IV Activity Assay

In total, 5 × 10^4^ Caco-2 cells/well were seeded in black 96-well plates with clear bottom. The second day after seeding, the spent medium was discarded and cells were treated with 100 μL/well of BUO and OMN extracts at concentrations of 10, 100, 500, or 1000 μg/mL or vehicle (C) in growth medium for 24 h at 37 °C. Experiments were carried out following previously optimized conditions [26]. More details are available in the Supplementary Material.

### 2.6. Computational Methods

The structures of the 6 simulated phenolic compounds were built using 2 different strategies. Hydroxytyrosol, tyrosol, oleacein, and oleocanthal were downloaded from PubChem, while Ole and La were first downloaded from PubChem in their glucoside form and then manually modified to their aglycone form using the Molecular Editor functions of the VEGA suite of programs. The obtained structures were then minimized by using AMMP as implemented in the VEGA environment [27].

Among the resolved DPP-IV structures, this study involved the complex between the enzyme and the inhibitor 75 M, PDB ID: 5T4F, chosen due to its very high resolution (1.90 Å). After deleting water molecules ions and crystallographic additives, the selected dimer was completed by adding the hydrogen atoms to amino acid residues and ionizing the protein considering physiologic pH at 7.4. The H++ server was chosen to predict the protonation state of ionizable protein groups. The protein was then refined by 2 minimization procedures using NAMD [28]. The first procedure was carried out with all the protein atoms fixed except for hydrogens; the second was performed with the backbone atoms under constraints to preserve the resolved folding. The prepared protein structure underwent the following docking simulations.

Docking simulations were carried out using PLANTS (Protein-Ligand ANT System) [29], which generates reliable ligand poses using the ant colonization algorithm (ACO). In detail, the search was focused on a 10 Å radius sphere around the bound ligand 75 M, thus including the entire binding cavity. For all the simulated ligands, PLANTS was used with default settings and without geometric constraints, speed 1 was used, and 100 poses were generated and scored by using the ChemPLP function. The obtained poses were evaluated considering both the docking scores and the involved residues.

### 2.7. Statistical Analysis

All the data sets were checked for normal distribution by the D’Agostino and Pearson test. Since they are all normally disturbed with *p*-values < 0.05, we proceeded with statistical analyses by one-way ANOVA followed by Tukey’s post-hoc tests and using Graphpad Prism 9 (San Diego, CA, USA). Values were reported as means ± standard deviation (S.D.); *p*-values < 0.05 were considered to be significant.

## 3. Results

### 3.1. BUO and OMN Extracts Inhibit the In Vitro and Cellular DPP-IV Activity

**Figure 1:**
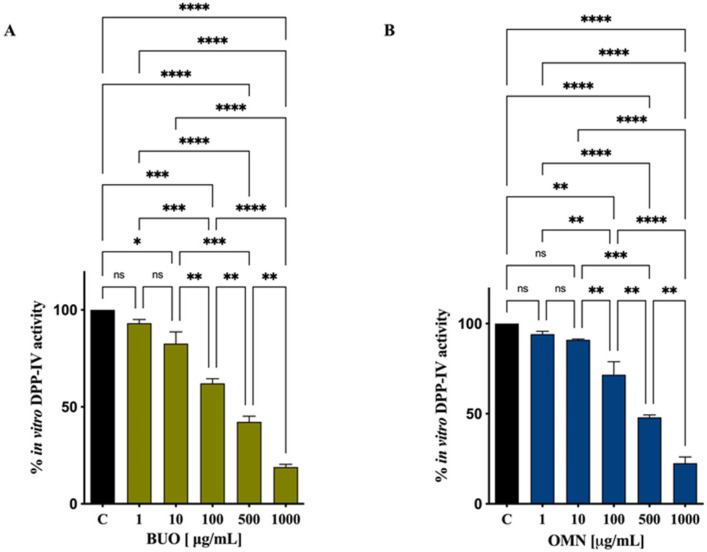
Effect of BUO (**A**) and OMN (**B**) on the in vitro DPP-IV activity. The data points represent the averages ± SD of 4 independent experiments performed in triplicate. All data sets were analyzed by one-way ANOVA followed by Tukey’s post-hoc test. ns: not significant; C: control sample (H2O). (*) *p* < 0.5; (**) *p* < 0.01; (***) *p* < 0.001; (****) *p* < 0.0001.

To evaluate the DPP-IV inhibitory activity of both BUO and OMN, in vitro experiments were performed by using the purified human recombinant DPP-IV enzyme and H-Gly-Pro-AMC as a substrate. The reaction was monitored by measuring the fluorescence signals at 465 nm after excitation at 350 nm, deriving from the release of a free AMC group after the cleavage of H-Gly-Pro-AMC catalyzed by DPP-IV. Figure 1A,B indicates that both extracts significantly reduced the DPP-IV activity in vitro: BUO reduced the DPP-IV activity by 6.8 ± 1.9, 17.4 ± 6.1, 37.9 ± 2.4, 57.8 ± 2.9, and 81 ± 1.4%, respectively (Figure 1A), at 1, 10, 100, 500, and 1000 µg/mL, whereas OMN reduced activity by 5.4 ± 1.7, 8.9 ± 0.4, 28.4 ± 7.2, 52 ± 1.3, and 77.5 ± 3.5%, respectively, at the same concentrations (Figure 1B).

**Figure 2:**
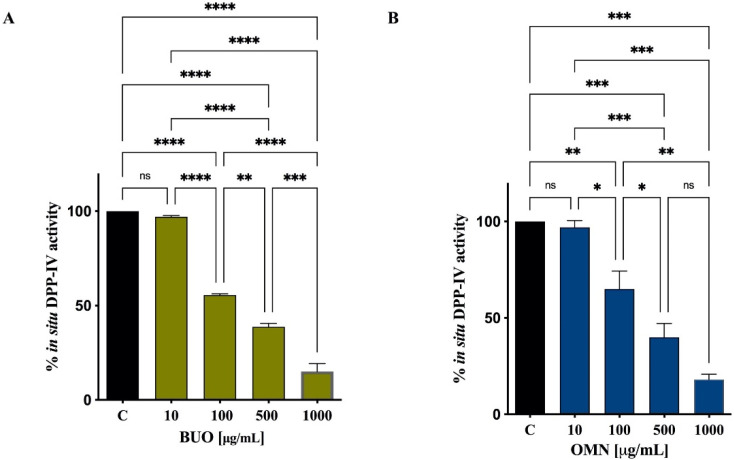
Effect of BUO (**A**) and OMN (**B**) on the cellular DPP-IV activity. The data points represent the averages ± SD of 4 independent experiments performed in triplicate. All data sets were analyzed by one-way ANOVA followed by Tukey’s post-hoc test. ns: not significant; C: control sample (H2O). (*) *p* < 0.5; (**) *p* < 0.01; (***) *p* < 0.001; (****) *p* < 0.0001.

Then, the DPP-IV inhibitory activity was assessed in cellular experiments using Caco-2 cells, which are an improved tool for the screening of DPP-IV inhibitors, since they are a reliable model of intestinal epithelial cells and express high levels of DPP-IV [30]. Since at in vitro level the concentration of 1 µg/mL was only slightly effective with regard to DPP-IV activity reduction, we decided to test only the concentrations of 10, 1000, 500, and 1000 µg/mL at cellular level. By monitoring the same fluorescent reaction, clear DPP-IV inhibitory effects were observed on Caco-2 cells also, as shown by Figure 2A,B. Notably, BUO reduced the cellular DPP-IV activity by 2.9 ± 0.7, 44.4 ± 0.7, 61.2 ± 1.8, and 85 ± 4.2% at 10, 100, 500, and 1000 µg/mL, respectively (Figure 2A), and OMN reduced the activity 3 ± 1.9, 35 ± 9.4, 60 ± 7.2, and 82 ± 2.8% at 10, 100, 500, and 1000 µg/mL, respectively (Figure 2B).

### 3.2. Analysis of Secoiridoids in BUO and OMN Extracts

To investigate on the concentration of some specific secoiridoids in BUO and OMN, ^1^H-NMR was used to quantitate four typical molecules that characterize the fresh EVOOs: oleacein, oleuropein, oleocanthal, and ligstroside aglycone. Using the manuscript of Karkoula et al. [23], the signals of the aldehydic protons of these secoiridoids were identified and their integral used for determining their percent concentration in the sample. This was possible by adding maleic acid as internal standard, that has a singlet at 6.29 ppm, in a range of the spectrum without interfering signals of the sample. The results shown in Table 1 were obtained applying the formula described in Section 2.2.

As for the ^1^H-NMR, only the use of CDCl_3_ guaranteed an adequate resolution to allow the quantitative evaluation of the selected secoiridoids. Furthermore, to avoid a perturbation of the chemical shifts of the target signals after the addition of the ISTD, it was necessary to add a very low volume of the maleic acid solution dissolved in CH_3_CN.

### 3.3. Assessment of the In Vitro Inhibition of DPP-IV Activity by Main EVOO Phenols

**Figure 3:**
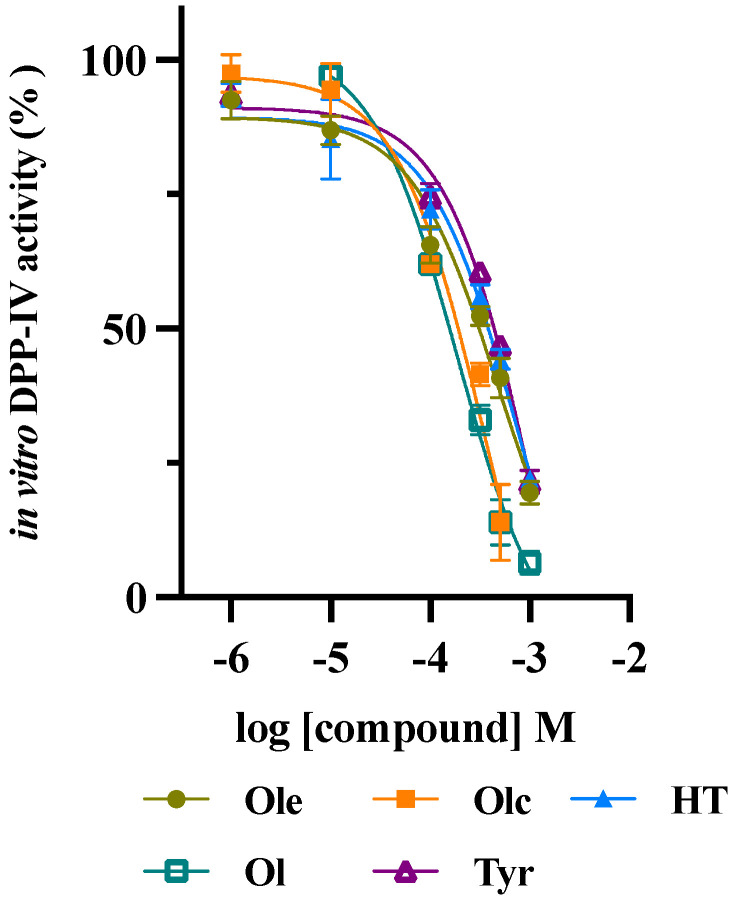
Dose—response effect of oleuropein (Ole), oleocanthal (Ol), oleacein (Olc), and hydroxytyrosol (HT), and tyrosol (Tyr) on the in vitro DPP-IV activity. The data points represent the averages ± SD of 3 independent experiments performed in triplicate.

Based on the EVOO phenol extract composition, it was decided to investigate the effect of the main secoiridoids on the in vitro DPP-IV activity. Therefore, oleuropein, oleacein, oleocanthal, hydroxytyrosol, and tyrosol were tested in the range of concentrations 1–1000 µM. Results clearly suggested that all the compounds inhibited the enzyme activity with a dose–response trend and IC_50_ values equal to 472.3 ± 21.7, 187 ± 11.4, 354.5 ± 12.7, 741.6 ± 35.7, and 1112 ± 55.6 µM for oleuropein, oleocanthal, oleacein, hydroxytyrosol, and tyrosol, respectively (Figure 3).

### 3.4. Molecular Docking Investigation

To better investigate the mechanism of action that lies beneath the DPP-IV inhibitory activity of BUO and OMN, a molecular docking investigation of their main phenolic components was carried out. As this study was performed a posteriori, the objective was not to obtain a prediction but instead a rationalization of the observed inhibition activity by analyzing the binding mode of the interaction with DPP-IV. Ligstroside aglycone was not tested in vitro, since it is not commercially available. However, due to its similarity with oleuropein aglycone, one may assume that this phytochemical also shares the DPP-IV inhibitory activity of the other secoiridoids.

**Figure 4:**
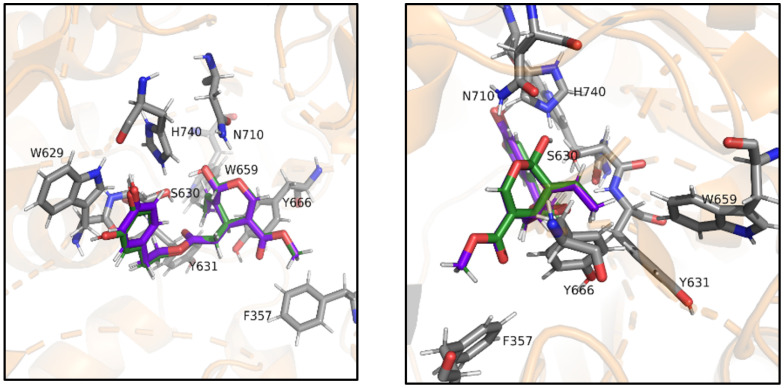
Putative complex between DPP-IV and oleuropein aglycone or ligstroside aglycone. Oleuropein aglycone (dark green) and ligstroside aglycone (purple) in complex with DPP-4, seen from the front (top) and the back (bottom). Key residues S630 and H740 of the catalytic triad appear to be evidently involved in the binding of both ligands.

Figure 4 shows the putative complexes that DPP-IV stabilizes with oleuropein aglycone or ligstroside aglycone, which appear fully superimposable. In detail, the aromatic rings of both molecules establish π-π stacking interactions with Trp629, while Ser630 is involved in H-bonds with both the carbonyl group of oleuropein aglycone/ligstroside aglycone and the hydroxy function of the dihydropyran ring. This hydroxy group is also involved in an H-bond with His740. Moreover, the ethylidene moiety of the dihydropyran is placed within a hydrophobic pocket lined by residues Tyr631, Trp659, and Tyr666 with which it stabilizes *p-p* stacking interactions. The heterocyclic oxygen atom establishes an H-bond with Asn710 and the carboxymethyl group further stabilizes the binding through apolar interactions with Phe357.

**Figure 5:**
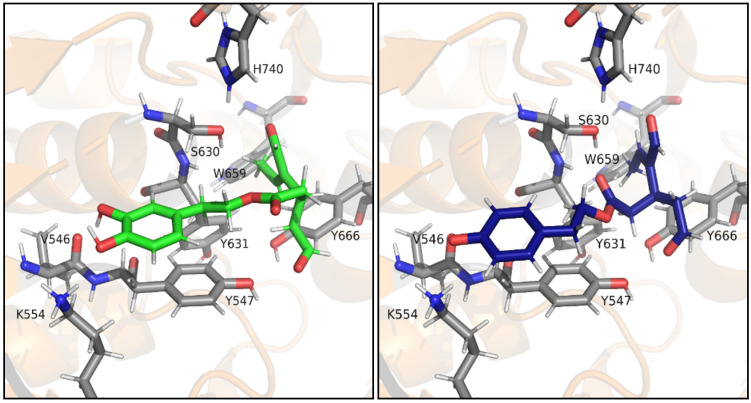
Putative complex between DPP-IV and oleacein or oleocanthal. Oleacin (blue) and oleocanthal (light green) in complex with DPP-4. Key residues S630 and H740 of the catalytic triad appear to be evidently involved in the binding of both ligands.

Figure 5 shows the binding of oleacein and oleocanthal to DPP-4. The two poses are less superimposed than before, as a consequence of the higher flexibility of these dialdehydes compared to the parent secoiridoids. The catechol and phenol rings partially overlap and are responsible for the π-π stacking interactions with Tyr547. Moreover, the phenolic functions establish H-bonds with the carbonyl group of Val546 and with Lys554. Ser630 is involved in an H-bond either with the aldehydic carbonyl oxygen atom of oleacein or with the ester carbonyl oxygen atom of oleocanthal. In both ligands, the ethylidene group is located within the hydrophobic pocket formed by Tyr631, Trp659, and Tyr666 and contributes to the binding as described above. For both oleacein and oleocanthal an aldehyde carbonyl oxygen atom is involved in an H-bond with Tyr547. Moreover, since oleocanthal interacts with Ser630 with the ester carbonyl, the remaining aldehyde establishes an H-bond with His740.

**Figure 6:**
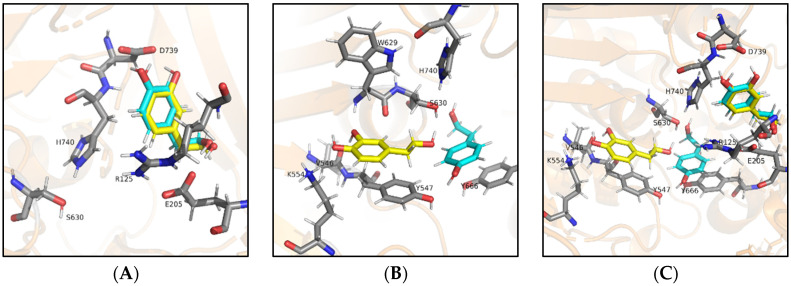
Putative complexes between DPP-IV and hydroxytyrosol or tyrosol. Hydroxytyrosol (yellow) and tyrosol (cyan) in complex with DPP-4. Binding mode A in subsite S2 (**A**) and binding mode B (**B**), involving key residue S630 of the catalytic triad. Overall view of both binding modes (**C**) for an easy comparison.

Figure 6 displays the binding modes of hydroxytyrosol and tyrosol. Indeed, due to their smaller size, two putative binding modes appear worth mentioning. In Figure 6A both hydroxytyrosol and tyrosol bind within the S2 subsite, and their alcoholic hydroxy group establishes an H-bond with Glu205. The binding of both ligands is further stabilized by the π-cation interaction between the aromatic rings and Arg125, while the catechol hydroxy groups and the phenolic hydroxyl establish H-bonds with Asp739. Figure 6B shows the other possible binding mode in which hydroxytyrosol displays the same binding mode previously described for oleacein and oleocanthal. In detail, the catechol ring establishes π-π stacking Tyr547 and the catechol hydroxy groups are engaged in H-bonds with Val546 and Lys554. The alcoholic hydroxy group of hydroxytyrosol interacts with Ser630. On the other hand, the pose for Tyr involves other residues seen before for larger ligands. The phenol ring establishes π-π stacking interactions with the Tyr666 while the phenolic hydroxyl is involved in an H-bond with Tyr547. At the same time, the alcoholic hydroxyl of Tyr is engaged in an H-bond with Ser630. Figure 6C provides an overall view of the two binding modes of hydroxytyrosol and tyrosol within the binding site of DPP-IV.

## 4. Discussion

Recently, the effect of EVOO and oleuropein on the post-prandial glycemic profile was investigated in randomized studies performed on healthy and pre-diabetic subjects. These studies revealed reduced activity and plasma levels of the enzyme DDP-IV and an improvement of the GLP-1 and GIP hormones, responsible for the release of insulin after food ingestion. With DPP-IV being upregulated in the presence of oxidative stress, the observed diminished activity and plasma levels after the consumption of EVOO or oleuropein have been rightly attributed to their antioxidant effect [12,13]. These findings prompted us to investigate whether a direct inhibition of the enzyme concomitantly occurred.

Notably, our results indicate that both BUO and OMN directly modulate the in vitro and cellular DPP-IV activity with a dose–response behavior. In detail, the in vitro DPP-IV activity assay was performed at the concentrations of 1, 10, 100, 500, and 1000 μg/mL, showing a statistically significant and dose-dependent inhibition by 6.8 ± 1.9, 17.4 ± 6.1, 37.9 ± 2.4, 57.8 ± 2.9, and 81 ± 1.4% for BUO and by 5.4 ± 1.7, 8.9 ± 0.4, 28.4 ± 7.2, 52 ± 1.3, and 77.5 ± 3.5% for OMN, respectively (Figure 1A,B).

Generally, in the area of food bioactive compounds able to inhibit DPP-IV activity, the investigations are carried out exclusively using biochemical tools based on the purified enzyme [31,32,33]. This traditional approach represents a great limitation for a more realistic characterization of the hydrolysates with DPP-IV inhibitory activity. In light of these observations, a specific feature of our work was the employment of an intestinal cell-based assay for measuring the enzymatic activity, which represents a complementary and cost-effective strategy for a more efficient discovery of food-derived DPP-IV inhibitors [26,30]. The enterocyte luminal surface expresses a great quantity of DPP-IV and for this reason, human intestinal Caco-2 cells represent a reliable tool for characterizing food derived DPP-IV inhibitors. Therefore, with the concentration of 1 μg/mL being only slightly effective in vitro, both BUO and OMN in the range of concentrations between 10 and 1000 μg/mL were assessed at cellular levels.

Findings suggested that BUO and OMN caused a reduction in DPP-IV activity by 2.9 ± 0.7, 44.4 ± 0.7, 61.2 ± 1.8, and 85 ± 4.2% and by 3 ± 1.9, 35 ± 9.4, 60 ± 7.2, and 82 ± 2.8% at 10, 100, 500, and 1000 µg/mL, respectively (Figure 2A,B), corroborating the in vitro results. In fact, comparing the in vitro (Figure 1) and cellular (Figure 2) data, the activity of both extracts is very similar, suggesting that both phytocomplexes may be stable to the metabolic activity of Caco-2 cells. Moreover, although these extracts were characterized by slightly different total phenolic contents, with OMN being richer, and by a different quantitative composition in the secoiridoid profile, with BUO being richer than OMN in hydroxytyrosol derivatives such as oleocanthal and oleacein [17], in in vitro and cellular experiments no statistically significant differences were observed between the EVOO phenolic extracts.

In addition, our findings are in line with previous work that demonstrated that a grape seed procyanidin extract (GSPE) can directly inhibit DPP4 activity [34]. In particular, GSPE inhibits in vitro DPP-IV activity by 54.67 ± 0.7 at 100 µg/mL. It seems therefore possible to affirm that in vitro the behavior of BUO and OMN extracts is similar to that of GSPE, whereas the EVOO phenolic extracts are more active than GSPE on human intestinal Caco-2 cells. In fact, while GSPE reduces the activity of the cellular enzyme by only 7.45 ± 1.5% at 100 µg/mL, BUO and OMN are 6- and 5-fold more active at the same concentrations on the same cellular model [34]. This different behavior may be explained by the different metabolic stabilities of the phytocomplexes in the presence of intestinal cells or by different affinities to the DPP-IV enzyme expressed on the Caco-2 cell membranes. In fact, GSPE is more active on the plasmatic and salivary DPP-IV than on the isoform, which is expressed on the membrane of Caco-2 cells [34].

To better describe the chemical characteristics of the two samples, the concentration of specific secoiridoids typically abundant in fresh EVOOs was evaluated by quantitative ^1^H-NMR. The use of HPLC-DAD to evaluate the effects of the acid hydrolysis on the extracts provides an interesting indirect estimation of the total secoiridoid forms produced by the transformation of the precursors, namely oleuropein and ligstroside (Table 1). The values obtained by ^1^H-NMR did not consider all the secoiridoid forms in the samples but were useful to confirm that BUO was richer in oleuropein derivatives (oleacein and oleuropein aglycone), in agreement with the results of the HPLC-DAD analyses. From the ^1^H-NMR spectra, it was possible to highlight a high concentration of the ligstroside aglycone, which is more than five times higher in OMN than in BUO.

In parallel, the effects of oleuropein, oleocanthal, oleacein, hydroxytyrosol, and tyrosol, the main secoridoids within both extracts, on the in vitro DPP-IV activity were analyzed. Results indicate that all the compounds inhibited the enzyme activity with a dose-response trend, with IC_50_ values equal to 472.3 ± 21.7, 187 ± 11.4, 354.5 ± 12.7, 741.6 ± 35.7, and 1112 ± 55.6 µM, respectively (Figure 3). Although all the compounds are active in the high micromolar range, oleocanthal is the most active, while hydroxytyrosol and tyrosol are less active.

However, so far only a few phenolic compounds have been shown to inhibit DPP-IV activity. Notably, it has been demonstrated that some flavonoids show an interesting ability to modulate the glucose homeostasis through the inhibition of DPP-IV activity. Indeed, luteolin, apigenin, quercetin, curcumin, kaempferol, and resveratrol reduce in vitro enzyme activity, with IC_50_ values ranging from 0.6 ± 0.4 nM (resveratrol) to 10.36 ± 0.09 μM (eriocitrin) [35]. In addition, it was demonstrated that curcumin is more active than resveratrol [36], whereas rutin, narirutin, naringin, hesperidin, limonin, neohesperidin, genistin, catechin, epicatechin, chlorogenic acid, and protocatechuic acid are totally inactive against DPP-IV activity.

The binding pose of resveratrol and curcumin in the DPP-IV active site, obtained by molecular docking, showed that they interact closely with key residues of sites S1, S2, and S3 within the active pocket of the enzyme [35,36] and that they act as competitive DPP-IV inhibitors, while luteolin and apigenin bound to DPP-IV in a noncompetitive manner.

Hence, in order to better explain these results and understand the mechanism of action of these extracts, an in silico molecular docking investigation was performed. Indeed, Figure 4 and Figure 5 show the binding of the bigger ligands oleuropein aglycone, ligstroside aglycone, oleacein, and oleocanthal that interact with the amino acidic residues present in the binding pocket and are cited in the literature as part of subsites S1, S1′, and S2′ [37] or Site 1 and 2 [38], especially Ser630 and His740 of the catalytic triad, thus preventing the endogenous peptide from collocating in the binding pocket and being cleaved by the protease. Being smaller molecules, hydroxytyrosol and tyrosol possess two different possible binding modes (Figure 6A,B).

Binding mode A (Figure 6A) is interesting because the ligands interact, among others, with residue Glu205 of the S2 subsite, which is usually filled with the protonated *N*-terminus (called residue P2) of the endogenous peptide and represents the anchoring site of the peptide substrate. According to this docking pose, the presence of either hydroxytyrosol or tyrosol in this subsite impedes the binding and cleavage of the peptide even if the catalytic triad is not involved.

Binding mode B (Figure 6B), on the other hand, is in line with the binding mode of the bigger ligands and involves the residues of the catalytic triad, preventing the substrate from binding and being cleaved. The possible existence of two different binding modes by hydroxytyrosol and tyrosol with the enzyme could explain the higher value of IC_50_ for these compounds, suggesting that binding mode A may be less efficient in blocking DPP-IV activity than binding mode B, which is the same for all the other compounds tested.

Apart from the fact that literature is very scarce with regard to phenolic DPP-IV inhibitors, an important aspect of these docking simulations lies in the fact that the putative binding of these molecules is built on hydrogen bonds and van der Waals interactions. Such an interaction pattern is very different from the binding mode of marketed DPP-IV inhibitors, which is characterized by a protonated amino group which stabilizes ion pairs with Glu205 and Glu206, mimicking what occurs for the endogenous peptide. This is well-mirrored by the IC_50_ values, which are in the high micromolar range, and thus very far from those of synthetic inhibitors that all share IC_50_ values in the low nanomolar range. However, these results emphasize that a weak but non-negligible inhibition can be induced even without eliciting those ionic interactions which are considered mandatory for the DPP-IV inhibition.

## 5. Conclusions

Thanks to a combination of biochemical, cellular, and computational techniques, this study for the first time provides relevant and innovative insights regarding the direct inhibition of DPP-IV by phenolic EVOO extracts. In addition, for the first time, we have demonstrated the inhibitory effects of the main secoridoids (oleuropein, oleocanthal, oleacein, hydroxytyrosol, and tyrosol) against DPP-IV activity. Possibly, the presence of less abundant biophenols within each extract, i.e., apigenin and luteolin, may synergistically contribute to the biological effect. Overall, these results allow us to explain the molecular mechanism of action through which EVOO contributes to post-prandial glycemia modulation.

## Figures and Tables

**Table 1:** Concentration of the 2 secoiridoids derived by oleuropein (Olc and Ole) and those derived by ligstroside (Ol and Lig); the data are compared with the amount determined by HPLC-DAD after acidic hydrolysis [17], and express the total content of the oleuropein derivatives and ligstroside derivatives as total hydroxytyrosol (HT) and total tyrosol (Tyr), respectively.

µg/mg Dry Extracts						
	^1^H-NMR				HPLC-DAD After Hydrolysis	
	Olc	Ol	Lig	Ole	Tot HT	Tot Tyr
BUO	88.4	77.1	86.2	156.2	208.0	156.0
OMN	69.7	135.2	466.8	297.5	151.0	275.1

## Data Availability

The data presented in this study are available in the results section.

## Supplementary Materials

The following are available online at https://www.mdpi.com/article/10.3390/antiox10071133/s1, Material and Methods Section.
