# Supplementary material for: Phenolic Extracts from Extra Virgin Olive Oils Inhibit Dipeptidyl Peptidase IV Activity: In Vitro, Cellular, and In Silico Molecular Modeling Investigations
Source: Antioxidants (Basel). 2021 Jul 16;10(7):1133. doi: 10.3390/antiox10071133 (PMC8301156; doi:10.3390/antiox10071133)
Supplement: Supplementary file 1 [file antioxidants-10-01133-s001.zip › antioxidants-1273106-supplementary.pdf]

Supplementary Materials

Phenolic Extracts from Extra Virgin Olive Oils Inhibit Dipeptidyl Peptidase IV Activity: In Vitro, Cellular, and In Silico Molecular Modeling Investigations

Carmen Lammi [1,*], Martina Bartolomei [1], Carlotta Bollati [1], Lorenzo Cecchi [2], Maria Bellumori [2], Emanuela Sabato [1], Vistoli Giulio [1], Nadia Mulinacci [2] and Anna Arnoldi [1]

[1] Department of Pharmaceutical Sciences, University of Milan, 20133 Milan, Italy; martina.bartolomei@unimi.it (M.B.); carlotta.bollati@unimi.it (C.B.); emanuela.sabato@studenti.unimi.it (E.S.); giulio.vistoli@unimi.it (V.G.); anna.arnoldi@unimi.it (A.A.)

[2] Department of Neuroscience, Psychology, Drug and Child Health, Pharmaceutical and Nutraceutical Section, University of Florence, 50019 Florence, Italy; lo.cecchi@unifi.it (L.C.); maria.bellumori@unifi.it (M.B.); nadia.mulinacci@unifi.it (N.M.)

* Correspondence: carmen.lammi@unimi.it; Tel.: +39-025-031-9372

2. Material and Methods

2.1. Chemicals

Tris-HCl, ethylendiaminetetraacetic acid (EDTA) and NaCl were from Sigma-Aldrich (St. Louis, MO, USA) The DPP-IV assay kit was from (Cayman Chemicals (Michigan, USA), while Gly-Pro-amido-4-methylcoumarin hydrobromide (Gly-Pro-AMC) was from AnaSpec (Freemont, CA, USA). Dulbecco's modified Eagle medium (DMEM), fetal bovine serum (FBS), L-glutamine, phosphate buffered saline (PBS), penicillin/streptomycin, 24 and 96-well plates were from Euroclone (Milan, Italy), polycarbonate filters, 12 mm in diameter, 0.4 mm in pore diameter, were from Transwell Corning Inc. (Lowell, MA, USA). Tyrosol (≥98%), hydroxytyrosol (≥98%), and oleocanthal (≥95%) were from Merck Life Science S.r.l. (Milano, Italy); oleuropein (≥98%) was from Extrasynthese (Genay, France), whereas oleacin was from Phytolab (Vestenbergsgreuth, Germany).

2.3. In Vitro DPP-IV Activity Assay

The DPP-IV enzyme was provided by Cayman Chemicals (Michigan, USA), while the DPP-IV substrate (H-Gly-Pro-AMC) was from AnaSpec Inc. (Freemont, CA, USA). The experiments were carried out in triplicate in a half volume 96-well solid plate (white). Each reaction (50 µL) was prepared adding the reagents in a microcentrifuge tube in the following order: 1 X assay buffer (30 µL) [20 mM Tris-HCl, pH 8.0, containing 100 mM NaCl and 1 mM EDTA], BUO and OMN EVOO extracts at final concentrations of 1, 10, 100, 500 and 1000 µg/mL or vehicle (C) or standard oleuropein, hydroxytyrosol, tyrosol, oleacein and oleocanthal (1-1000 µM) (10 µL), and finally the DPP-IV enzyme (10 µL). Subsequently, the samples were mixed and 50 µL of each reaction was transferred in each plate well. Each reaction was started by adding 50 µL of substrate solution to each well and incubated at 37° for 30 minutes. Fluorescence signals deriving from the release of free AMC were measured using a Synergy H1 fluorescence plate reader from BioTek (excitation/emission wavelength 350/465 nm respectively).

2.4. Cell Culture

Caco-2 cells, obtained from INSERM (Paris, France), were routinely subcultured at 50% density and maintained at 37°C in a 90% air/10% CO_2 atmosphere in DMEM containing 25 mM of glucose, 3.7 g/L of $NaHCO_3$, 4 mM of stable L-glutamine, 1% non-essential amino acids, 100 U/L of penicillin and 100 μg/L of streptomycin (complete medium), supplemented with 10% heat-inactivated fetal bovine serum (FBS; Hyclone Laboratories, Logan, UT, USA).

2.5. In Situ DPP-IV Activity Assay

A total of 5 x 10^4 Caco-2 cells/well were seeded in black 96-well plates with clear bottom. The second day after seeding, the spent medium was discarded and cells were treated with 100 μL/well of BUO and OMN extracts at the concentration of 10, 100, 500, 1000 μg/mL or vehicle (C) in growth medium for 24 h at 37°C. Afterwards, treatments were removed and Caco-2 cells were washed once with 100 μL of phosphate buffered saline (PBS) without Ca^{2+} and Mg^{2+}, before the addition to each well of 100 μL of Gly-Pro-AMC substrate at the concentration of 50.0 μM in PBS without Ca^{2+} and Mg^{2+}. Fluorescence signals deriving from the release of free AMC were measured using a Synergy H1 fluorescence microplate reader from BioTek (excitation/emission wavelength 350/465 nm respectively) every 1 min for up to 10 minutes.
